# Driving the life course approach to vaccination through the lens of key global agendas

**DOI:** 10.3389/fragi.2023.1200397

**Published:** 2023-07-31

**Authors:** Andra Stancu, Anusheh Khan, Jane Barratt

**Affiliations:** International Federation on Ageing, Toronto, ON, Canada

**Keywords:** life-course immunization (LCI), adult vaccination, older adults, national immunisation program, global agenda

## Abstract

Globally, our population is ageing at an unprecedented rate and by 2030, which marks the end of the United Nations (UN) Decade of Healthy Ageing, the number of people aged 60 years and older will be 34% higher than today, reaching 1.4 billion. Vaccination is one of the most effective public health interventions of modern times and a key action in fostering healthy ageing throughout the life-course. To promote wellbeing at all ages, global agendas including the WHO Immunization Agenda 2030, the UN Decade of Healthy Ageing and the World Health Organization (WHO) Global Report on Ageism outline strategic actions and guidance to help implement policies and programs. Yet, the linkages between healthy ageing, functional ability and adult vaccination are not substantively recognized or integrated as cross-cutting themes, which impacts operationalization into national immunization plans. When aligned and connected strategically, these agendas have potential to substantially contribute to policy change to prioritize life-course immunization and support the preservation of function at all stages of life. This article describes the intersecting goals and visions of these strategic agendas and identifies specific elements of overlap, which when connected, could strengthen the development of comprehensive and effective national immunization policies.

## Introduction

Globally, population is ageing at an unprecedented rate and by 2030, which marks the end of the United Nations (UN) Decade of Healthy Ageing. The population of adults over the age of 60 years will outnumber adolescents and young adults. Specifically, the population of older adults will increase 34% by the year 2030 in relation to current data, and more than double to 2.1 billion individuals by 2050. (United Nations, Department of Economic and Social Affairs, Population Division, 2017; World Health Organization, 2022a).

Parallel to population ageing is the increase in prevalence of non-communicable diseases (NCDs). NCDs kill 41 million people each year which is equivalent to 74% of all deaths worldwide (World Health Organization, 2022b). Although people of all ages are affected by NCDs, the burden is higher among older adults due to the cluster of risk across the lifetime. In developed nations, about one in four adults have at least two chronic medical conditions, and more than one-half of all older adults have three or more conditions (Sangster and Barratt, 2021). In Europe, cardiovascular diseases, diabetes, cancer, chronic respiratory diseases and mental disorders together represent approximately 77% of the disease burden and 86% of total deaths (Sangster and Barratt, 2021). In the Americas, NCDs including cancer, cardiovascular disease, chronic respiratory illness and diabetes are responsible for seven out of ten deaths among people aged 70 years and older (Pan American Health Organization, 2023).

Ageing is also associated with the remodeling of immune changes and defined as immunosenescence (Xu et al., 2020). There are three main hallmarks of immunosenescence, namely, the reduced ability to respond to new antigens, the accumulation of memory T cells and the state of low-grade chronic inflammation “inflamm-aging”) (Aiello et al., 2019). Immunosenescence contributes to an increased incidence, morbidity and mortality of infections (Agrawal and Weinberger, 2022).

Vaccination is one of the most effective public health interventions of modern times and a key action in fostering healthy ageing throughout the life-course (from childhood to adulthood) (Kuruvilla et al., 2018). In addition to health benefits, vaccination also reduces burden on healthcare systems and has a significant return on investment, both for the individual and the society, when considering the alternatives including high medical costs, loss of daily function and independence, lost productivity of the individual and potential caregivers, and exacerbation of chronic conditions (Privor-Dumm et al., 2021). Several vaccines are recommended specifically for older adults including COVID-19, influenza, pneumonia, shingles, and pertussis (Privor-Dumm et al., 2021). Yet, the uptake rates of adult vaccination remain suboptimal among at-risk populations and the life-course approach to immunization is not implemented in all countries as illustrated by National Immunization Programs (NIPs) that are largely pediatric focused (Eiden et al., 2023).

The COVID-19 pandemic has further highlighted not only the disproportionate impact of vaccine-preventable disease (VPDs) on older people but also considerable shortcomings in healthcare systems and policies at multiple levels of decision-making (National Institute on Ageing, 2022). There is a critical need to ensure integrated policy development across traditionally siloed sectors and intergovernmental agencies to strengthen healthcare including health promotion through prioritization of national life-course immunization policies.

For more than a decade, the World Health Organization (WHO) has urged health systems to improve health outcomes and drive economic and social stability (Organisation for Economic Co-operation and Development, 2022). The WHO Immunization Agenda 2030 (IA 2030), the UN Decade of Healthy Ageing (the Decade) and the WHO Global Report on Ageism independently and collectively represent generational defining agendas with the potential to improve the health of citizens across the life course (World Health Organization, 2019a; World Health Organization, 2020a; World Health Organization, 2021). Each of these agendas call on Member States to promote wellbeing for all ages by responding to the WHO Thirteenth General Programme of Work 2019–2023 (GPW13) triple billion targets: one billion more people benefiting from universal health coverage; one billion more people better protected from health emergencies; and, one billion more people enjoying better health and wellbeing (World Health Organization, 2019b). From a broad perspective, each of these agendas outline strategic actions and guidance to promote health and wellbeing for all ages. Yet, the linkages between healthy ageing, functional ability and adult vaccination are not substantively recognized or integrated as cross-cutting themes, which impacts operationalization into national immunization plans. When aligned and connected strategically, these agendas have potential to substantially contribute to policy change to prioritize life-course immunization and support the preservation of function at all stages of life.

This article describes the intersecting goals and visions of these strategic agendas and identifies specific elements of overlap, which when connected, could strengthen the development of comprehensive and effective national immunization policies.

### Goals of global agendas in the context of healthy ageing and life-course immunization

The WHO defines healthy ageing as the process of developing and maintaining functional ability that enables wellbeing in older age (World Health Organization, 2019c). Functional ability is determined by our intrinsic capacity (the sum of all physical and mental capacities) and the interaction between the individual and the environments. In the conceptual framework of the life-course defined by the WHO, ‘functional ability’ and ‘intrinsic capacity’ are shown diagrammatically as idealized curves across the life-course. When considering the population as a whole, functional ability and intrinsic capacity can vary across the second half of the life-course and general trajectories can be divided into three common periods: relatively high and stable intrinsic capacity; declining capacity, and significant loss of capacity (World Health Organization, 2020c). Immunization as an effective public health action has individual, community and societal value across all stages of later life when functional capacity can range from high stable capacity, to declining capacity to significant loss of capacity (World Health Organization, 2020c). The focus of public health strategies including immunization should be on maintaining high and stable levels of intrinsic capacity for as long as possible. Therefore, expanding the focus of vaccination from childhood to vaccination across the life-course is crucial to healthy ageing.

Global agendas including the IA2030, the WHO Global Report on Ageism, and the UN Decade of Healthy Ageing individually speak to the importance of fostering functional ability and contribute to the overarching goals of the GPW13 by promoting an integrated approach to health. However, there are significant shortcomings in developing the case for life-course immunization.

The IA2030 is a global strategy that positions immunization as a contributor to the fundamental right to enjoyment of the highest attainable physical and mental health. It is positioned as an investment in the future in three ways: saving lives and protecting the health of populations, improving the productivity and resilience of countries, and ensuring a safer and more prosperous world. The IA2030 specifically focuses on increasing access and uptake of immunization and recognizes the benefits of vaccination across the life-course (World Health Organization, 2020a). However, despite this recognition, IA2030 emphasizes childhood vaccination and in comparison pays little attention to older adults who are not only the largest growing global population but also at high-risk of serious complications from VPDs. For example, IA2030 notes that “vaccines prevent disability, which can impair children’s growth and cognitive development … ” but fail to acknowledge and address the significant and often long-term functional decline experienced by older adults following a serious infection (World Health Organization, 2020a).

The WHO Global Report on Ageism outlines a framework for action to reduce institutional, interpersonal and self-directed ageism and focuses on promoting the health and wellbeing of older adults through a society-wide approach. It calls for a more equitable approach to healthcare for older adults and recognizes the need to address their unique health needs and challenges (World Health Organization, 2021). However, in the bigger conversation about ageism and health, a key consideration is not only the intentions but also the outcomes. For example, policies in the health sector that allow an intervention to be rationed by age would be considered as a form of institutional ageism. In the context of life-course immunization, for example, higher national targets for childhood vaccination compared to adult vaccination could be argued as ageist. Age discrimination in vaccine prioritization is shown to be embedded in wider ageist attitudes in health policy which may give the lives of older people a lower social value than the lives of people at younger ages, and is currently not accounted for in the Global Report on Ageism. Therefore, combatting ageism in the policy dialogue on life-course immunization is a critical component of transforming health systems to prioritize health prevention and promotion.

The UN Decade of Healthy Ageing is a global collaboration that seeks to reduce health inequities and improve the lives of older people, their families and communities by action in four areas: combatting ageism, integrated care, long-term care and age-friendly environments (World Health Organization, 2020b). Aligned with the IA2030 and Global Report on Ageism, the Decade aims to promote the health and wellbeing of older adults through a society wide approach and ensure healthcare decisions affecting older persons respect their dignity and promote human rights, including the right to enjoy the highest attainable standard of health (World Health Organization, 2020b). Although the Decade calls for transformation of health systems away from disease based curative models, little consideration is given to lifestyle behaviors and health promotion with the exception of the mention of risk factors associated with NCDs. Moreover, lack of attention is paid to adult vaccination and ‘vaccines’ is only mentioned once alongside medical products and technologies in the context of ensuring availability and access. The narrative connecting VPDs, vaccination and function, and therein creating an environment that enables older people to do what they have reason to value is not prominent.

### Aligning global agendas to implement the life-course approach to vaccination

The term “life-course” is used liberally in global and national agendas. The main outcome of a life-course approach to health in the context of the UN Decade of Healthy Ageing and IA2030 is functional ability and a good quality of life. For this to be contemplated there is a need to strategically align and connect priorities across agendas (ageing, health, immunization) at a national level to inform a comprehensive national immunization plan that truly reflects vaccination throughout life.

Over the past decade, there have been numerous and consistent calls for action by civil society organizations to reorient health systems to equitable models that ensure health for all (World Health Organization, 2019c; Laurent-Ledru et al., 2011). The Immunization for All Ages (IFAA) manifesto is an international call for action to improve immunization infrastructure and increase investment in health promotion and preservation of function at all stages of life through a comprehensive immunization strategy. Seeking to create solutions to gaps in international agendas, the initiative calls for three actions critical to improving adult vaccination (International Federation on Ageing, 2021):

1) Prioritize immunization throughout life as a key pillar of expanded prevention strategies and a central component of universal health coverage.
2) Remove barriers to access for appropriate immunization throughout life to ensure all people are protected and no one is left behind.
3) Reduce inequities in timely, appropriate, and affordable access to immunization throughout life (International Federation on Ageing, 2021).

The IFAA is an advocacy lever among many such calls for action from civil society actors to raise awareness among Member States around the critical need to improve adult vaccination strategies. However, generating political will to action recommendations requires robust collaboration and may benefit from an explicit mandate to drive change at the national level. Governments must take stock of the repeated calls for action and prioritize evidence-based recommendations to respond to the needs of older adults at a national and global level.

To ensure the implementation of life-course immunization, it is critical to strategically align global agendas to substantially contribute to policy change. To improve alignment between agendas, strategies around healthy ageing must build the link between adult vaccination and functional ability and highlight the health, social and economic case of life-course immunization. The agendas must collectively highlight the critical need to integrate life-course immunization across health sectors, including primary and long-term care, and the involvement of multidisciplinary and cross-sectoral representation in immunization policy development. Moreover, global agendas should all speak to the importance of setting consistent vaccination coverage targets for all age groups and dedicating funding to ensure equitable access to vaccines such as eliminating out-of-pocket costs.

Aligning global agendas to push for a collective agenda on life-course immunization is essential to ensure operationalization in NIPs and policies.

## Conclusion

In the midst of unprecedented and sustained increase in population of people with diverse functional abilities, combined with increasing prevalence of NCDs and VPDs, there is a critical need to support healthy ageing through a long-term perspective on health system transformation to prioritize life-course immunization.

A life-course approach to vaccination is key to fostering healthy ageing and functional ability and has significant benefits by improving health outcomes, sustaining productivity, and enhancing economic development for all ages. Yet, this approach is not implemented through national policies and the uptake rates of adult vaccination remain suboptimal globally.

Establishing a life-course approach to vaccination as a valuable policy framework and implementation in national programs will require prioritization and building strategic connections across global agendas including the IA 2030, Global Report on Ageism and the Decade. Aligning the global agendas to reorient health systems to a life-course approach to vaccination will inevitably assist in reaching the triple billion targets.

## Data Availability

The original contributions presented in the study are included in the article/Supplementary Material, further inquiries can be directed to the corresponding author.
